# Recognition of Soccer Player Actions Using a Synchronized Multi-Camera and mm-Wave Radar Platform

**DOI:** 10.3390/s26082532

**Published:** 2026-04-20

**Authors:** Daniël Benjamin Keyter, Johan Pieter de Villiers

**Affiliations:** Department of Electrical, Electronic and Computer Engineering, University of Pretoria, Hatfield 0028, South Africa

**Keywords:** computer vision, FMCW radar, human action recognition (HAR), mm-wave radar, player tracking, radar–camera fusion, sensor fusion

## Abstract

This paper presents a multimodal sensing approach for fine-grained soccer action recognition using synchronized mm-wave FMCW radar and multiview RGB cameras. A TI IWR1443BOOST FMCW radar and three Sony IMX296 global-shutter cameras were used to record seven soccer-related actions in different movement directions in an outdoor environment. Range–Doppler radar processing is applied to extract global mel features and CFAR-localized block representations of mel and radar spectrogram features to capture both coarse and fine micro-Doppler characteristics. Camera features are derived from bounding box, HOG, optical flow, and pose estimations. Classification is performed using logistic regression as the classical model and various deep models. Performance is evaluated using cross-validation. Radar alone achieved moderate performance (0.897 F1_macro_ using TCN), successfully identifying coarse motion but showing limited separability for dribbling-based actions. Camera-only models achieve near-perfect accuracy (≥0.997 F1_macro_ using 1D-CNN), with the confusion matrices being nearly perfectly diagonal already. The best performance is obtained from a cross-modal transformer with multiple cameras (0.998 F1_macro_). These results demonstrate that a camera by itself performs strongly for the action recognition task but also that radar–camera fusion can improve robustness and enhance the discrimination of finer soccer player movements for outdoor analytics and player monitoring applications.

## 1. Introduction

Player tracking systems used in sports offer numerous potential benefits. For broadcasters, the use of a player tracking system may provide important contextual data that enhances the overall viewing experience. The system can generate statistics based on player movement and potentially player actions, which can be presented to viewers. Action recognition assists in identifying key moments in a match, enabling the creation of highlight packages after the game. Sports teams can leverage data to manage player load and gain insight into the impact of player positioning on match outcomes, leading to improved tactical decision-making. Integrating human action recognition (HAR) with player tracking has the potential to add additional context to the data that is being collected and can be used for better motion predictions and statistical player analyses.

While radar–camera fusion has been explored in prior work for human activity recognition and object detection, its application to fine-grained soccer-specific action recognition in outdoor environments remains limited.

A mm-wave radar as a sensor has the benefit of being resistant to weather effects, lighting, and occlusion, as well as being able to provide accurate range and radial velocity measurements. Radar HAR methods typically analyze time–frequency micro-Doppler spectrograms, which capture the Doppler shifts caused by moving limbs. Existing studies use hand-crafted features from these spectrograms. Javier and Kim apply linear predictive coding on the envelopes of the radar spectrograms for HAR [1]. This was achieved in an indoor environment using only radial movements with classes including walking, crawling, and boxing. The reported accuracy is over 85%, and it is noted that a long enough observation window is required to obtain high accuracies. Caglıyan and Gürbüz also used radar spectrogram envelopes for HAR using a BumbleBee Radar in an indoor environment [2]. The authors used a treadmill to keep the subject at a constant distance from the sensor, and they classified between walking, jogging, crawling, and crawling at a near tangential angle. The precision was reported between 0.837 and 0.94. Zhang et al. applied a Bayesian network on spectral data [3]. The dataset used was the Mellon University Graphics Lab Motion Capture Database [4]. The classes used are walk, run, and jump, and the reported classification accuracy ranged from 95.34% to 98.11%. Chae et al. combined range–Doppler and Doppler-time features to monitor head motions but did not provide numerical results [5]. Lin et al. performed HAR using mm-wave radar in an indoor environment by using feature fusion across time–frequency, range angle domains [6]. Classification was performed using CNN-BiLSTM and PCANet-based fusion. Combinations of certain actions were classified, and this includes bending, squatting, standing, walking, and falling with a reported accuracy of 99.75%.

Van Eeden et al. used mel-frequency cepstral coefficients (MFCCs) to distinguish humans from animals in the field [7]. A Gaussian mixture Hidden Markov model (GMM-HMM) approach was applied, and a classification accuracy between 75% and 90% was obtained when classifying between humans and different animals. Even though this paper does not focus on differentiating human actions, it still shows the potential of using MFCC data for motion classification in an operational environment. From the above existing research, it can be seen that radar-based HAR can achieve high accuracies for classifying coarse actions, but there is a lack of research on the more fine-grained actions that relate to sport, specifically soccer. Radar-based HAR can achieve high accuracy for coarse actions, but further research is needed for fine-grained soccer-specific motion.

Using a camera for HAR has the benefit of providing rich spatial and contextual information regarding a player’s pose and environment. Vision-based HAR is a well-documented topic where deep learning is often used to learn spatio-temporal features automatically. Zhang et al. recognized activities by performing multitask learning with the addition of attribute regularization on the KTH [8], UIUC [9], and Olympic Sport datasets [10], with their approach outperforming existing methods at the time [9]. Le et al. performed continuous action and gesture recognition using a sliding window approach to analyze hand motions over time. They report classification accuracies of 0.95, 0.97, and 0.71 on the IPN [11], UOW, and InHARD datasets [12], respectively, for isolated actions [12]. They did, however, report accuracies of only 0.57, 0.76, and 0.33 for continuous action classification on the same respective datasets.

Jeon et al. proposed a lightweight radar–camera fusion deep learning model for human activity recognition in a recent study [13]. Their model achieved a 98.74% classification accuracy on actions such as answering a phone, drinking, taking off glasses, grabbing a handle, sitting, standing, pickup, fall, recovery, handshake, walking, running, entering, and exiting. Yi et al. performed HAR using a multimodal fusion model that also utilizes both camera and radar data [14]. Multiple classes were defined, including hand movements, head movements, leg lifts, hand raises, squats, stooping motions, body twists, and walking. The system was tested in both normal and complex environments. Both of these studies include only a single co-located camera. The method obtained a reported average recognition accuracy of 99.3%. Keyter and de Villiers performed action recognition in an outdoor environment on a small dataset for camera and radar fusion to determine which features are potentially suitable for the task [15]. It was concluded that MFCC data for the radar and histogram of oriented gradients (HOG) features for a camera are potentially the most suitable features to use. They have classified four classes, namely walk, jog, dribble walk, and dribble jog.

Hu et al. performed pose estimation with radar as opposed to cameras to predict bone lengths and joint rotation angles by integrating deep learning with forward kinematics [16]. A mean joint error of roughly 3.5 cm was achieved. Using pose estimation from radar can lead to improved accuracy of HAR and could be beneficial in the soccer player action recognition sense. Rivas-Caceido et al. performed HAR using IMU sensors [17]. The data was collected by placing five IMUs on participants’ hands, knees, and chest. The sensors collected orientation, linear acceleration, and angular velocity information. They were able to classify multiple activities at an average accuracy of 93.5%. Even though this approach has some merit, it is too invasive for soccer player action recognition with the amount of sensors on multiple players, thus making it impractical.

Despite notable studies published on the broader subject of HAR, the studies are typically under ideal conditions and without the intricacies of soccer player action recognition. Measurements are taken in controlled or ideal environments, which include being indoors or the use of treadmills to keep the target within a certain range bin for micro-Doppler processing. The existing research also focuses on actions that are easier to separate, with actions such as kicks and dribbling being excluded. Dribbling with a soccer ball is very similar to normal walking or running, with the detail lying in the micro-Doppler signatures of these actions, which means the features need to be handled in such a way that these fine details can be used to separate between classes. Hence, it would be beneficial to perform research in an outdoor environment with these actions that are more difficult to separate to determine the degree to which it is possible to accurately classify soccer player actions.

The existing literature also tends to focus on radial movement with regard to radar. Range–Doppler processing provides radial velocity, and classification tasks become more challenging when the targets move tangential to the radar, which ultimately minimizes the data present in the Doppler domain. Owing to this challenge, it becomes crucial to focus on the micro-Doppler effects that can be captured using mm-wave radar.

The use of radar and camera sensors in a complementary manner could thus potentially leverage the strengths of each sensor while mitigating their shortcomings and ultimately improve soccer player action recognition. This is a field that has not been studied thoroughly. A synchronized multicamera and radar system will be used to create a dataset that can be used in further studies relating to this topic.

A conference paper by the authors [15] presented an exploratory study focusing primarily on the analysis of radar and vision feature representations using a single smartphone camera and a loosely synchronized sensing setup. The objective of that work was to investigate the behavior of different feature types for soccer-related actions. In contrast, the work presented in this paper addresses a different research objective, namely the development and evaluation of a synchronized multimodal sensing framework for fine-grained soccer player action recognition. The current study introduces a multiview RGB camera setup synchronized with mm-wave radar, enabling accurate temporal alignment between sensing modalities. Furthermore, the scope of this work has been substantially expanded to include multimodal feature fusion, ablation studies across sensor configurations, direction-dependent performance analysis, which includes tangential motions, and computational cost evaluation. Consequently, while the two studies are related in terms of application domain, the present paper focuses on multimodal action recognition and system-level evaluation rather than feature exploration.

The main contributions of this work are summarized as follows:

- Multimodal sensing framework: a synchronized radar–camera system, consisting of a single radar and multiple cameras at different positions, is developed for fine-grained soccer action recognition in an outdoor environment, capturing complementary motion and visual information
- Comprehensive feature analysis: a wide range of radar (micro-Doppler, mel-spectrogram, MFCC) and visual (HOG, optical flow, pose, bounding box) features are systematically evaluated.
- A block-based radar feature representation using CFAR-based region-of-interest selection and Doppler-centered alignment of the dominant return to improve the consistency of micro-Doppler signatures and enabling better separation of torso and limb motion.
- Extensive ablation and modality comparison: the contributions of individual feature groups and sensing modalities are analyzed through controlled ablation studies, including radar-only, camera-only, and fused configurations.
- Direction-aware evaluation: the impact of motion direction (e.g., radial, tangential, diagonal, and horizontal) on radar and fusion performance is investigated.
- Computational cost analysis: a detailed comparison of classical and deep learning models is provided, highlighting trade-offs between accuracy and efficiency for real-world deployment.

## 2. Materials and Methods

**Figure 1:**
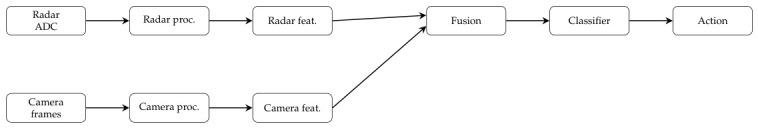
High-level overview of the proposed multimodal radar–camera action recognition pipeline.

In this section, we describe the proposed multimodal radar–camera action recognition pipeline consisting of data collection, radar processing and feature extraction, camera processing and feature extraction, temporal windowing and multimodal fusion, and classification and evaluation. An overview of the system can be seen in Figure 1.

### 2.1. Data Collection and Experimental Setup

**Figure 2:**
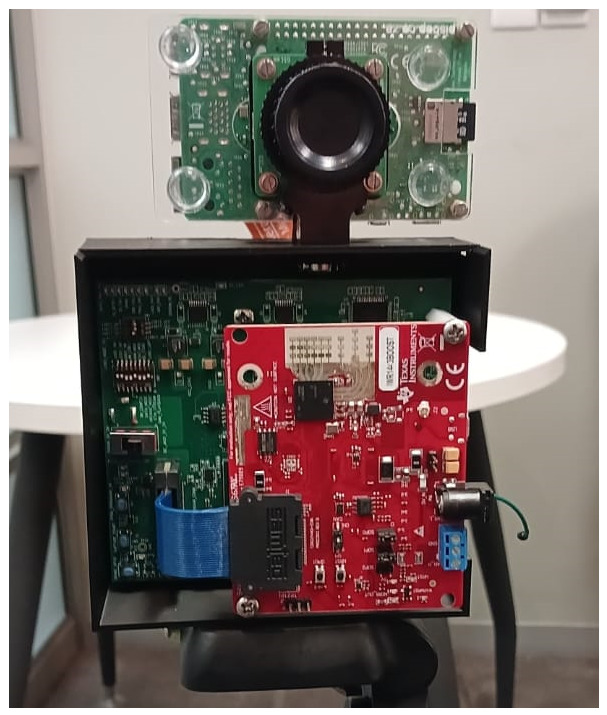
Semi-co-located radar–camera pair used for data collection.

Radar data was collected using the IWR1443Boost mm-wave FMCW radar and DCA1000EVM data capture board (Texas Instruments, Dallas, TX, USA). Each clip contains 500 radar frames, recorded at a slow-time sampling period of 40 ms. Three global-shutter cameras were deployed to provide complementary RGB viewpoints. Each camera used the IMX296 imaging sensor (Sony Corporation, Tokyo, Japan), a global shutter camera from the Raspberry Pi camera suite (Raspberry Pi Ltd., Cambridge, UK). The cameras recorded at 25 fps to match the slow-time capture rate of the radar. The cameras were each placed at chest height with one co-located with the radar on the horizontal axis as seen in Figure 2. The cameras are synchronized using software, and the radar is hardware synchronized to one of the cameras. Two participants with different body types and fitness levels were then tasked to perform actions related to soccer, as well as some used in the literature, that include walk, jog, dribble-walk, dribble-jog, jump, kick, and crawl. The difference in participants introduces subject variability in motion dynamics. Although the number of subjects is limited, additional variability is introduced through multiple repetitions and varying motion directions, which helps improve the robustness of the learned models. The actions were performed in different directions where the radar–camera pair is the reference point. These include radial, diagonal (45° and 135° from the radar–camera pair), horizontal, and tangential movements in both directions between the start and end points.

**Figure 3:**
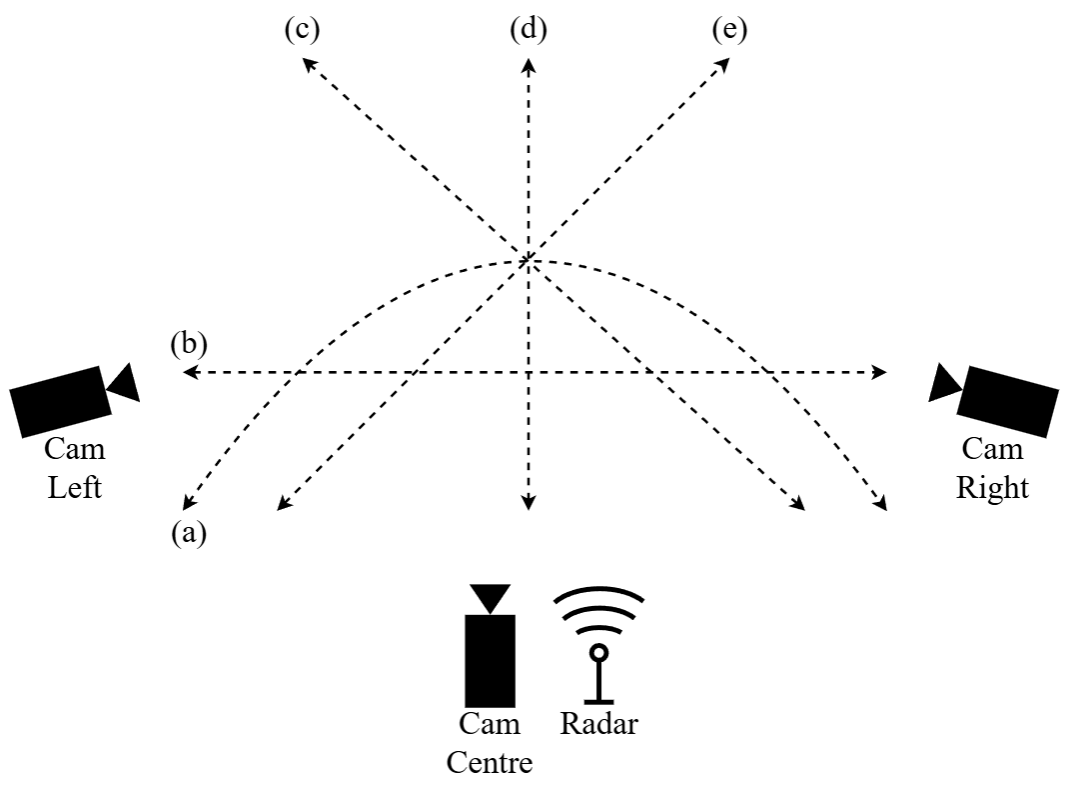
Placement of the three cameras and the radar along with the directions of the actions performed by the player, with (a) moving tangentially, (b) horizontally, (c) diagonally to the right, (d) radially, and (e) diagonally to the left. Note that the radar and center camera are co-located on the *x*-axis and that directions are relative to this sensor pair.

The placement of the cameras and radar, as well as the directions of motion, can be seen in Figure 3, while Table 1 describes the radar system specifications. Table 2 and Table 3 describe the dataset that was created.

### 2.2. Radar Processing and Feature Extraction

**Figure 4:**
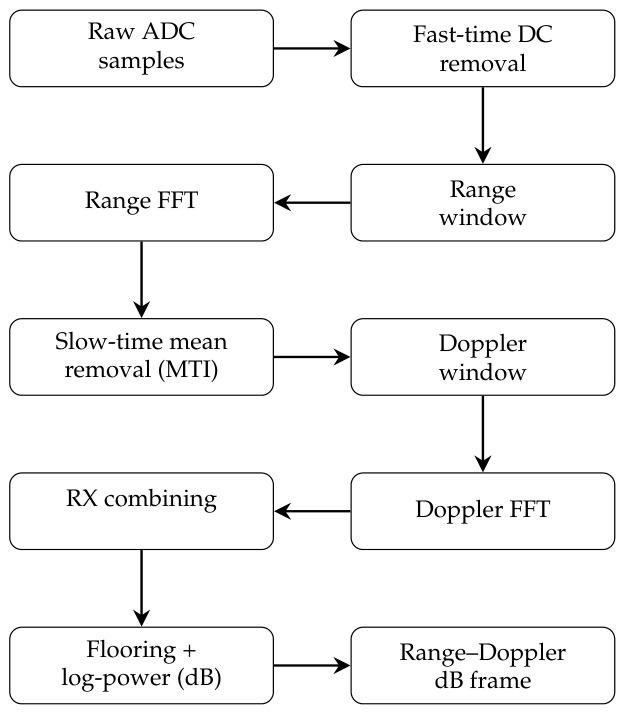
Radar signal processing pipeline from raw ADC samples to log-power range–Doppler (RD) frames.

Figure 4 illustrates the processing chain used to obtain log-power range–Doppler frames ready for feature extraction from the raw ADC samples. The pipeline consists of fast-time DC removal, range windowing and FFT, slow-time mean removal (MTI), Doppler windowing and FFT, RX combining, and finally log-power conversion.

The radar front-end employs an FMCW architecture with a direct-conversion quadrature receiver, producing complex baseband signals where the in-phase (I) and quadrature (Q) components encode amplitude as well as phase information. While ideal receivers assume perfect orthogonality between these channels, practical systems exhibit amplitude and phase imbalance due to hardware imperfections. This leads to a distortion of the IQ constellation from a circular to an elliptical trajectory and introduces artefacts such as spectral distortion, ghost targets, and mirrored components in the micro-Doppler signature as described by Cardillo [18].

In this work, range–Doppler processing is performed directly on the raw IQ data using a standard two-stage FFT pipeline. A fast-time FFT is applied along each chirp to obtain the range information, followed by a slow-time FFT across chirps to extract Doppler information. Before this, the data is conditioned using DC offset removal, static clutter suppression, windowing, and non-coherent integration across receive antennas to improve robustness under real measurement conditions.

No explicit hardware-level IQ calibration is applied. Instead, the classification models are trained on real measured data, allowing residual hardware non-idealities to be handled implicitly within the learning process.

#### 2.2.1. CFAR-Based Block Localization

Given that the target does not remain within a specific range bin, as is often the case in the literature, the extended human target needs to be detected to generate the radar features. A CFAR-like detector is applied to each range–Doppler frame to achieve this [19]. For every cell (d,r), a local noise estimate is computed using a 2D training window with guard cells removed. A detection is declared when

(1) $$R_{\mathrm{dB}}\left[t,d,r\right]>T\left[d,r\right]+K,$$

where T[d,r] is the local mean and *K* is a constant offset. A 3×3 non-maximum suppression filter retains only local peaks, producing a sparse detection mask.

A window of size (H×W) is slid across the RD frame. Each location is scored using a combination of RD amplitude and detection-mask activity. The window with the maximum score defines the block location $\left(y_{t}^{*},x_{t}^{*}\right)$, and the corresponding block

(2) $$\begin{array}{cc} B_{t}\left[d,r\right] & =R_{\mathrm{dB}}\left[t,\;y_{t}^{*}+d,\;x_{t}^{*}+r\right],\\ \; & \;\;\;d\in \{0,\ldots ,H-1\},\;r\in \{0,\ldots ,W-1\}, \end{array}$$

is used for the block-level Doppler processing and for the per-range and block-based radar feature extraction described next. To stabilize the representation across frames, the block is re-centered such that the maximum-power Doppler bin (typically corresponding to torso motion) is aligned to the block center. This improves separability between torso motion and surrounding limb-induced micro-Doppler signatures.

#### 2.2.2. Radar Feature Extraction

Radar features are derived from both the full range–Doppler map and the CFAR-selected block $B_{t}$. This results in three complementary representations: global Doppler features, block-level features, and per-range extended-target features. Together, these provide both coarse and fine-grained motion descriptors for the extended human target.

Global features summarize Doppler activity across all range bins and capture overall motion intensity. Block-level features restrict the analysis to a compact Doppler neighborhood around the torso bin, which produces the strongest and most stable radar return, with the torso centered to reduce temporal drift. Limb movements, such as those from arms and legs, generate additional micro-Doppler components that vary more rapidly and are distributed around the torso motion. Without recentering, these components may shift across Doppler bins due to variations in target motion, leading to inconsistent representations over time. By aligning the dominant Doppler component to the block center, the torso motion is stabilized, and the limb-induced micro-Doppler signatures become more symmetrically distributed around it. This improves the separability between the central (torso) and peripheral (limb) motion components and is expected to lead to more consistent and discriminative features for classification. Per-range features preserve the spatial structure of the extended human target by extracting a Doppler signature independently for each range column, enabling the model to separate torso motion from limb-induced micro-Doppler at neighboring ranges.

The complete set of radar features is summarized in Table 4.

All mel and MFCC features are computed using standard mel-filterbank processing and a DCT-II transform. For each frame *t*, all radar descriptors (global mel, global MFCCs, global mel energy, block mel, block MFCCs, center Doppler, and per-range mel) are concatenated into a single radar feature vector. These frame-level vectors are aggregated over temporal windows for multimodal fusion.

**Figure 5:**
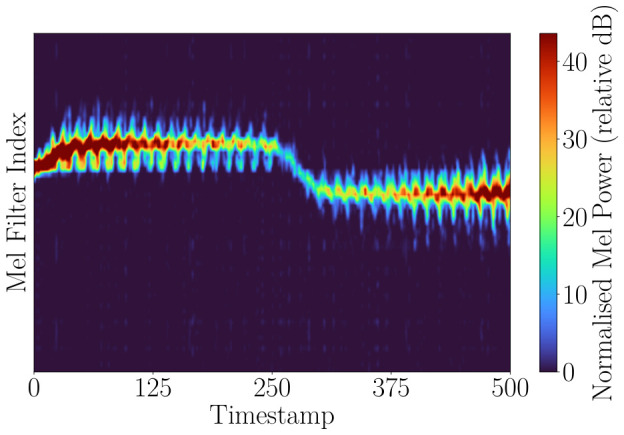
Global mel spectrogram computed from the full Doppler spectrum for a walking human. The dominant torso return is clearly visible; however, finer limb-induced micro-Doppler components are less distinguishable due to the global nature of the representation, spectral smoothing introduced by the mel transformation, and the underlying Doppler resolution. This representation provides an overall motion overview but does not explicitly separate individual motion components.

**Figure 6:**
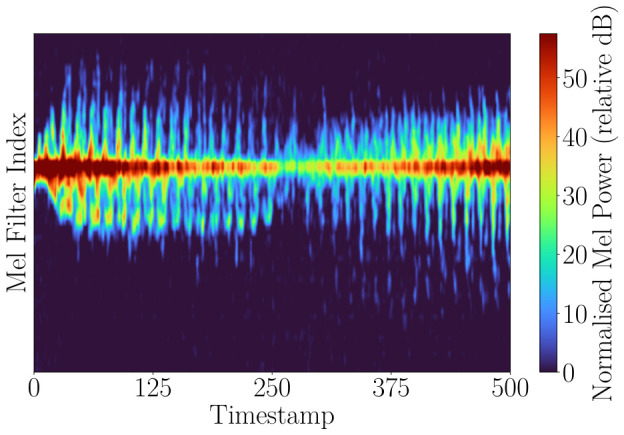
Block mel spectrogram corresponding to the detected region of interest for a walking human. The region is centered on the dominant Doppler component, reducing background interference and enhancing the visibility of limb-induced micro-Doppler variations compared to the global representation in Figure 5.

Figure 5 shows the global mel spectrogram computed from the full Doppler spectrum. Due to the dominance of the torso return and the inclusion of background components, finer micro-Doppler signatures associated with limb motion are less clearly separated. In addition, the effective resolution is influenced by radar processing parameters, such as the number of Doppler bins and FFT configuration, which further limit the ability to resolve closely spaced motion components. Figure 6 shows the mel spectrogram computed from a localized block centered on the detected region of interest. By focusing on this region and aligning the dominant Doppler component, background interference is reduced, and limb-induced micro-Doppler variations become more pronounced. This representation should therefore be more suitable for capturing fine-grained motion characteristics. To further improve the representation, the detected region is recentered such that the maximum-power Doppler component is aligned to the block center. This stabilizes the dominant torso return over time and provides a consistent reference point. Without this alignment, changes in target velocity shift the Doppler signature, which can blur the micro-Doppler patterns. By fixing the dominant return, limb-induced variations remain centered and become easier to distinguish. Figure 6 shows the block-based mel representation.

### 2.3. Camera Processing and Feature Extraction

Camera-based features complement the radar modality by capturing appearance, motion, and articulated pose information. A summary of the camera features used can be seen in Table 5. All features except full-frame HOG are computed from the player-centered region of interest (ROI) obtained from YOLO-based person detections [22]. The pose, extracted from the ROI using the OpenCV library [23], is normalized to a fixed body size to ensure scale- and translation-invariant articulation features.

Optical flow and pose estimation provide complementary motion representations that enhance the multimodal framework. Optical flow captures dense, pixel-level motion between consecutive frames, enabling the modeling of fine-grained movements such as limb dynamics. In contrast, radar captures motion via micro-Doppler signatures, providing robust measurements of radial velocity along the line of sight.

Pose estimation further contributes a structured representation of human body dynamics by encoding joint-level relationships. While radar reflects the underlying motion dynamics, pose features provide additional spatial context that is not directly observable from radar alone. These representations offer complementary perspectives: radar captures velocity-based motion information, optical flow captures detailed motion fields, and pose estimation captures high-level structural information.

Recent advances in optical flow estimation, such as PWC-Net [24], and transformer-based approaches for pose estimation [25] highlight the increasing capability of deep models to capture complex motion patterns. These developments further support the role of vision-based representations in modeling human motion within multimodal systems.

**Table 5:** Camera feature groups extracted per frame.

Feature Group	Description
Bounding Box	Player center (x,y), width, height, and aspect ratio.
HOG (Full)	Histogram of oriented gradients over the full image [26].
HOG (ROI)	HOG descriptor within the player bounding box.
Optical Flow	Dense optical-flow magnitude and orientation statistics [27].
Pose Keypoints	Normalized 2D joint coordinates.

**Figure 7:**
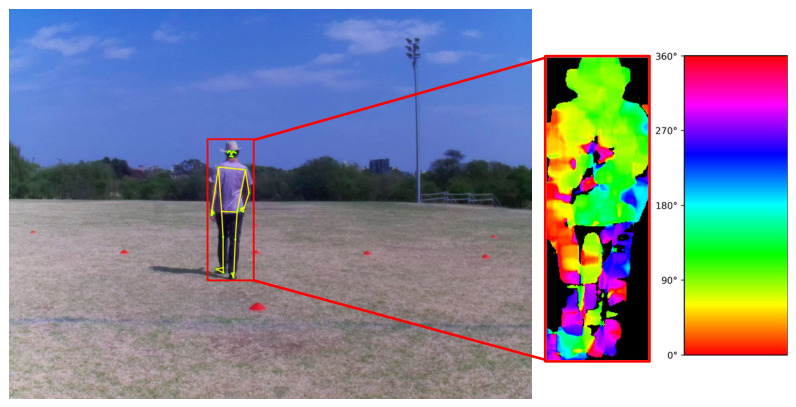
The left part of the image displays the bounding box and wireframe of the subject projected onto a frame from the video captured by the center camera. The optical flow visualization within the detection box for the same video can be seen on the right with the legend indicating the direction. Hue encodes motion direction with 0° corresponding to the right of the image, and increasing angles move in a counter-clockwise direction. Brighter colors indicate more movement, and black indicates a lack of motion.

Figure 7 displays the bounding box, pose estimation and optical flow visualization of a player.

### 2.4. Temporal Windowing and Multimodal Fusion

Radar and camera features are temporally aligned by frame, ensuring that each timestamp contains a corresponding set of radar and camera descriptors. The features are aggregated into temporal windows of 192 frames, which corresponds to 7.68 s at 25 FPS. A 50% overlap is used to preserve continuity between windows.

Three separate fusion strategies are evaluated. Early fusion concatenates radar and camera features along the feature dimension within each window. Pooled statistics serve as input to an L1-regularized logistic regression, while the raw fused sequence is leveraged directly by the gated recurrent network (GRU), long short-term memory (LSTM), 1D-CNN, and temporal convolution network (TCN) models. Late fusion trains separate radar-only and camera-only logistic regression classifiers independently, combining their predicted probabilities via a weighted sum, with weights (radar: 0.2, camera: 0.8) selected via a sweep over the validation set. From doing a sweep of hyperparameters, it was noted that reducing the large per-range features from radar negatively affects the fusion results if not reduced, hence these features are collapsed for fusion, while the full features are used when only radar is available.

### 2.5. Classification and Evaluation Protocol

A classical and a deep model are used for classification. Features were standardized and projected using principal component analysis (PCA) [28], retaining 95% of the total variance, and split using the GroupKFold cross-validation implementation provided by scikit-learn (Version 1.3.2) [29] over 5 folds (roughly 80% training/20% testing per fold) to avoid temporal leakage. Experiments were conducted with a fixed random seed of 42 for cross-validation, PCA, PyTorch (Version 2.4.1, CUDA 12.1), NumPy (Version 1.24.4), and Python (Version 3.8.20) random to ensure reproducibility. The classical model used is logistic regression [30]. For deep sequence modeling, five architectures are evaluated to span the space of temporal modeling approaches. GRU [31] and LSTM [32] are employed as representative recurrent architectures capable of capturing long-range sequential dependencies, with bidirectional processing to exploit both past and future context within each window. A 1D-CNN [33] is included as a computationally efficient convolutional baseline that captures local temporal patterns. TCN [34] extends this approach with dilated causal convolutions and residual connections, providing an exponentially larger receptive field without the vanishing gradient challenges associated with recurrent architectures. Finally, a cross-modal transformer (CMT), built on the self-attention mechanism of Vaswani et al. [35], is included as the sole fusion-aware architecture for radar–camera fusion. Its dual-stream encoder with bidirectional cross-attention is designed to model interactions between the radar and camera streams, making it the only classifier in this study that explicitly learns inter-modality relationships rather than treating the fused feature vector as a single undifferentiated input. Table 6 shows the architecture specifications for the chosen classifiers, and Table 7 shows the hyperparameters chosen for the deep classifiers.

Given that the dataset is unbalanced, classification accuracy alone is not a sufficient metric. Macro F1, balanced accuracy (bAcc), precision, recall, and confusion matrices are used to represent the performance of the classifiers.

## 3. Results

Table 8 displays the classification results for radar on all classes in all directions when only a single feature is used to indicate which features work well for classification. The same is displayed for the combination of all cameras in Table 9. Table 10 displays ablation study results when all features are used with a single feature set omitted. These tests are performed without the use of PCA to avoid mixing information across feature dimensions and to preserve the direct interpretability of individual feature groups. Applying PCA prior to ablation would obscure the contribution of individual feature dimensions and introduce an additional optimization variable. This does, however, include the risk of contamination by feature groups of high dimensionality, and it is expected that using PCA will lead to an improved accuracy in the final classification results. These tests are performed using all of the camera sensors.

Table 11 shows the results obtained for the different movement directions in Figure 3 when only using radar to evaluate the effect of the different motion angles relative to the radar boresight on the classification accuracy. This excludes jumping and kicking. Classification is performed for both the classical and deep learning approaches. Table 12 compares the classification results of the different individual sensors and a subset of possible sensor combinations for a single modality, while Table 13 compares the results where radar and camera features are fused. These results include all actions and results mentioned previously without any exclusions. These tests are performed with PCA being applied on the features for logistic regression with an explained variance of 95%.

**Figure 8:**
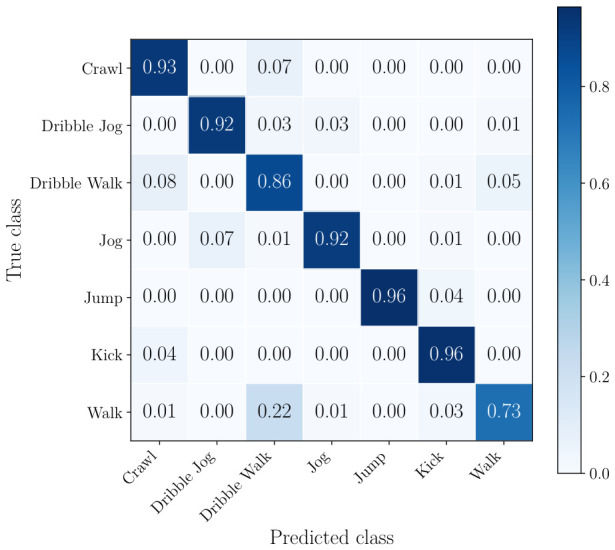
Confusion matrix for radar-only classification.

**Figure 9:**
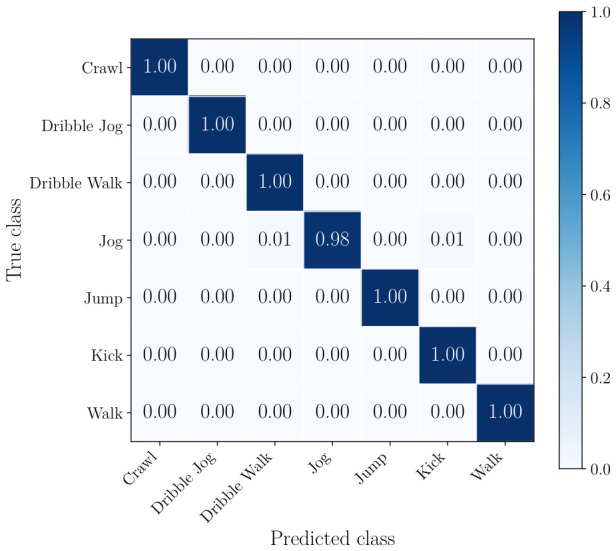
Confusion matrix for camera-only classification using the right camera.

**Figure 10:**
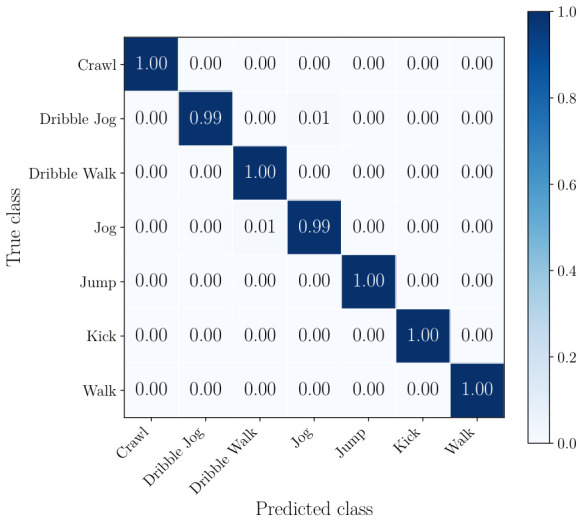
Confusion matrix for classification using sensor-fusion for the center and right camera as well as the radar.

The normalized confusion matrices of the radar, the best performing camera, and the best combination of sensors can be seen from Figure 8, Figure 9 and Figure 10. This includes all classes.

**Figure 11:**
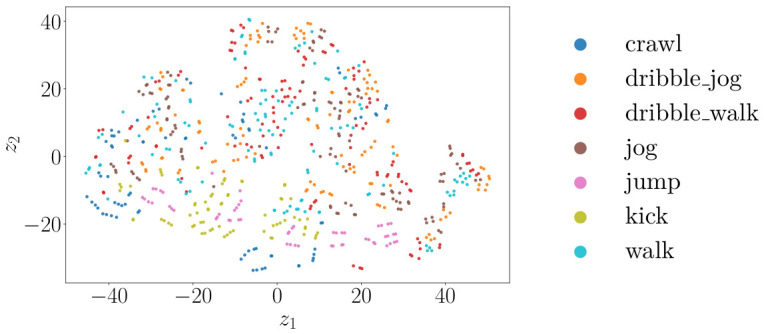
t-SNE scatterplot for radar features.

**Figure 12:**
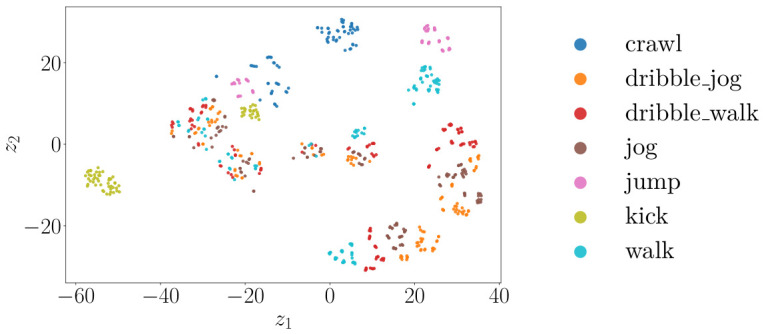
t-SNE scatterplot for camera features.

**Figure 13:**
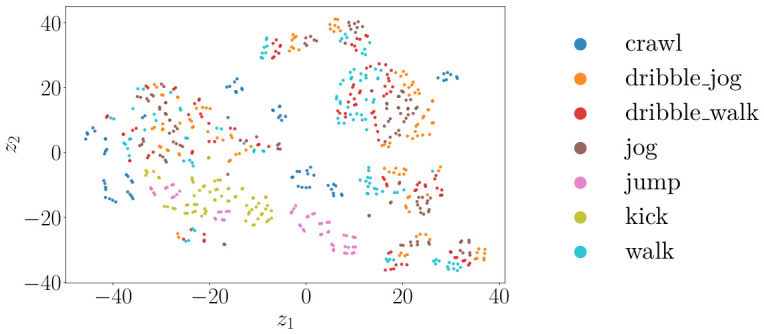
t-SNE scatterplot for radar–camera fusion features.

Figure 11, Figure 12 and Figure 13 show the t-SNE [38] embeddings of the window-level features for the radar, camera, and best performing fused modalities. The legend corresponds to all classes in the relevant plots.

Table 14 displays the computational cost of processing raw radar and camera data, and Table 15 displays the computational cost of the feature extraction. The computational cost is in milliseconds and FPS. Table 16 shows the computational cost of the classification across modalities. The cost of PCA, logistic regression, and 1D-CNN is presented in seconds.

## 4. Discussion

Table 8 and Table 9 indicate that certain feature groups demonstrate a strong standalone performance, suggesting that they encode motion patterns directly relevant to action discrimination without requiring complementary modalities. This is especially true for the camera features, with the HOG features for the entire frame performing the best, followed by the HOG features for the bounding box around the soccer player. Conversely, several feature groups show limited standalone performance, suggesting that they are either weak or require supplemental features for classification. This is the case with several radar features. For the center Doppler of the targets, which relates to the main body sway and the mel energy, the classification accuracy was low. The spectrogram for the detected bounding box in the radar spectrogram achieved the highest standalone accuracy amongst the radar features, indicating that it could be a strong feature. It should also be noted that the strongest standalones also tend to be the features with the higher dimensionalities. In this test, the classical logistic regression achieved lower accuracies for all feature groups except for the HOG of the full frame, where it achieved the highest accuracy overall.

The ablation study in Table 10 indicates that the omission of the HOG features for the full frame results in the most significant drop in performance in terms of the acc and bAcc metrics, with the omission of the HOG features for the region of interest showing the second most significant drop in performance. This corresponds to the data in Table 9. It should be noted that the omission of radar features does not lead to a significant decrease in classification performance and in some cases results in a slight improvement. This is due to the strong discriminative power of the visual features in the current dataset, where camera-based information dominates the classification task. In contrast, radar features provide complementary motion information, which becomes more relevant in challenging scenarios such as variations in motion direction or conditions where visual data may be degraded. A general observation is that in all of the cases, accuracy and F1_macro_ score drops are statistically modest, which suggests that discriminative information may be duplicated in multiple features and the dropping of any single feature has a minimal effect on performance. This points to robustness in the current selection of features.

From Table 11, it can be seen that the radial direction achieves the highest classification accuracy for the radar modality. This is expected, as FMCW radar measures radial velocity directly, and motions aligned with the radar line of sight produce strong, well-separated Doppler signatures. In contrast, horizontal and tangential motions exhibit much weaker radial components, leading to poorer Doppler separability and consequently lower recognition performance. This especially affects the accuracy of the logistic regression and GRU results. These classifiers also achieve poor accuracy for the horizontal motions, followed by the tangential motions. The diagonal motions produce intermediate results and are slightly poorer than the radial and combined motions. It does, however, outperform the combined case where the 1D-CNN is used. For all cases, with the exception of the combined motions, 1D-CNN achieves the best results, and deep models broadly outperform logistic regression across all directions, confirming that exploring temporal patterns within the 192-frame window is critical for radar-based action recognition. Compared to Table 8, there is an increase in the overall accuracy when the features are combined over the best case for a single feature group This table shows that the block spectrogram per range performs the best overall for radar and suggests that it is the strongest of the radar features. Table 9 shows that HOG features for the full frame are the strongest features by themselves, achieving a high accuracy for all classifiers. From Table 8, Table 9 and Table 12, it can be seen that radar by itself, though reasonable, does not classify nearly as accurately as a single camera. A single center camera under TCN achieves 0.993 ± 0.015 F1_macro_, compared to a maximum of 0.897 ± 0.033 for radar-only under the same classifier, a gap that persists across all deep architectures. Table 13 suggests that adding a second camera at a different position yields further improvement, with the right and center camera achieving 0.996 ± 0.008 under both 1D-CNN and TCN. Even though adding radar to a single camera performs worse than adding a second camera, it can still prove beneficial, given that you can use a single co-located radar–camera setup to gain a slight boost in accuracy. Interestingly, when radar is combined with two cameras, classification performance matches or exceeds the three-camera camera-only configuration for all of the deep models, providing direct evidence that radar features carry complementary information not captured by additional viewpoints alone. Adding a third camera does not improve the best performance but does benefit logistic regression for both early and late fusion and also slightly improves for the 1D-CNN.

Prior radar-only approaches typically report classification accuracies in the range of 85–90% under controlled indoor conditions [1,2], which aligns with the radar-only performance observed in this work. More recent radar-based methods employing advanced feature fusion and deep learning have reported significantly higher accuracies, exceeding 98% in some cases [6]. However, these results are generally obtained on constrained indoor datasets with relatively coarse activity classes and limited variability.

In contrast, camera-based methods often achieve near-perfect performance due to the richer spatial information available in visual data, particularly in controlled environments [9,12].

More recent multimodal radar–camera approaches report accuracies exceeding 98% by combining micro-Doppler and visual features [13,14]. However, these studies typically focus on relatively simple daily activities and are evaluated in controlled indoor environments, which limits the variability of the observed motion patterns.

In this work, the focus is on fine-grained soccer actions, which involve more complex motion patterns and object interactions. A GroupKFold strategy is also used to ensure proper separation between training and testing clips. Despite the increased task complexity, the proposed multimodal system achieves a macro F1-score of up to 0.998. Direct numerical comparison remains difficult due to differences in dataset characteristics and evaluation protocols.

In the confusion matrix in Figure 8, it is shown that the radar-only approach achieves over 0.85 per-class accuracy for all classes except walk. This class is most frequently misclassified as dribble walk, which is expected given the subtle differences in motion between these two activities and the limited micro-Doppler separation in radar data. Similarly, dribble walk is misclassified as crawl and walk and jog, but to a lesser extent. In Figure 9, it can be seen that using a single camera already mitigates most of the shortcomings of radar, with only jog being misclassified as dribble walk and kick on a small number of occasions. Figure 10 shows that the combination of two cameras and a radar classifies soccer player actions extremely accurately with minimal misclassification.

The t-SNE embeddings in Figure 11 display partially overlapping clusters for the radar, consistent with the misclassifications seen in Figure 8. Even though kick, jump, and crawl form distinct clusters, there is significant overlap for the walk, dribble walk, and dribble jog classes. In contrast, Figure 12 indicates that the camera features produce tighter, more distinct clusters, indicating that visual data has higher discriminative power for the activities performed. Even though there is still some overlap, it is to a significantly lesser extent. The fused representation exhibits the most clearly separated clusters. Even though the clusters appear less compact than in the embeddings for only using camera sensors, there is less overall overlap. These observations correspond to the reported classification results.

From the computational results reported in Table 14 and Table 15, it can be seen that the cost for radar computation is dominated by the loading of the data. This step is I/O-bound and depends primarily on storage throughput rather than computational complexity. Since the raw radar data files are large and stored on external media, data transfer time exceeds the time required for subsequent signal processing operations. Feature extraction steps incur negligible computational cost in comparison. CFAR detection incurs low computational cost due to the use of GPU processing in this case. Computing the mel filterbank and MFCC extraction incurred the highest cost of the features. Camera processing is detection-limited, with object detection accounting for the majority of the computational cost. Most of the features have relatively low computational times with the exception of pose estimation, which takes significantly longer than the other features that were computed. In the case of both modalities, feature extraction stages are computationally inexpensive and scale favorably. In the case of the radar, if data transfer rates could be improved, inference duration could be significantly reduced. Data processing can also be sped up by utilizing GPU processing for the creation of bounding boxes and pose estimation.

Table 16 shows that classical classification typically operates faster than deep models with the exception of the a single camera being used. The use of the full radar features also significantly slows down training for the radar-only case for the reasons given in Section 2.4. If the radar features are not collapsed, the computational cost for the radar-only case will be significantly lower than that of the fusion-based approaches. The evaluation times are, however, still similar.

## 5. Conclusions

This work investigates the effectiveness of combining mm-wave radar with multiview RGB cameras for fine-grained soccer player action recognition in an outdoor environment. By jointly analyzing global Doppler information and CFAR-localized extended-target representations, and integrating these with appearance-, motion-, and pose-based visual features, the proposed framework demonstrates that radar and cameras provide complementary and mutually reinforcing information for human action recognition.

The experimental results indicate that mm-wave radar is effective at capturing velocity-based motion characteristics, while camera data excels at distinguishing actions with subtle kinematic variations, such as dribbling versus normal walking or jogging. Although camera-only models achieve very high recognition performance under favorable conditions, radar–camera fusion consistently produces the most discriminative feature representations at the highest overall classification accuracy. This highlights the value of radar as an auxiliary sensing modality that enhances robustness, particularly in scenarios where visual sensing may be degraded by lighting, occlusion, or environmental factors.

The findings further indicate that even a single co-located radar–camera configuration can yield measurable gains, while combining radar with multiple camera viewpoints offers the strongest overall performance. While this study focused on a controlled set of soccer-specific actions and a limited number of subjects, the proposed framework provides a foundation for future work on more diverse player behaviors, larger-scale deployments, and more challenging environmental conditions.

These findings indicate a gap in current radar feature design for human action recognition, particularly in the development of features that capture articulated motion and temporal dynamics beyond global Doppler statistics.

The ablation results demonstrate that camera-based features consistently outperform radar-only features when evaluated in isolation, reflecting the rich spatial information captured by visual sensors. This performance gap is expected, as human actions are inherently defined by body pose and motion, which are directly observable in video but only indirectly inferred from radar reflections. These findings suggest that current radar feature representations do not yet fully exploit the information contained in range–Doppler measurements, highlighting a gap in radar feature design rather than a fundamental limitation of the sensing modality. Consequently, this work motivates further research into radar features that capture temporal motion patterns. Although radar achieves lower classification accuracy than camera-based modalities when used in isolation and does not yield substantial accuracy gains in fusion under ideal conditions, it provides a critical redundancy mechanism in scenarios where optical sensing is degraded. Camera performance is inherently susceptible to adverse environmental conditions such as poor lighting, motion blur, occlusion, and weather effects, all of which have no effect on FMCW radar operation. In such conditions, the radar stream can maintain classification capability independently, ensuring system robustness beyond what the ablation results under controlled conditions alone suggest. The radar-only classification results presented for radar in Table 12 consequently represent a minimum performance floor under complete camera failure, with the full multimodal system expected to operate significantly above this bound under normal conditions.

### Limitations and Future Work

The dataset currently includes soccer actions performed by only two participants. Although cross-validation was employed and windows from the same recording clip were kept within the same partition to avoid temporal leakage, the limited number of subjects may restrict the generalization of the model to players with different body characteristics, playing styles, and movement patterns. Future work will therefore focus on expanding the dataset to include a larger and more diverse group of participants.

Additionally, the actions considered in this study were performed in a structured and instructed manner to ensure consistent data collection across modalities. In real match scenarios, player movements are more dynamic and unpredictable, and visual occlusions or environmental variations may occur. These factors introduce a domain gap between the controlled experimental setting and real-world deployment. Future work will investigate the application of the proposed multimodal sensing approach in less controlled, real-world match environments.

Additional future work includes an extension to multiple targets and application to real footage from sports matches and analysis of results.

## Figures and Tables

**Table 1:** Radar system specifications.

Parameter	Value	Unit
Start Frequency	77	GHz
Sampling Rate	11	MHz
Chirp Duration	49	µs
Number of ADC Samples	512	samples
Number of RX Antennas	4	
Number of Tx Antennas	3	
Range Resolution	0.0805	m
Doppler Resolution	0.0881	m/s

**Table 2:** Overview of the multimodal dataset. Range–Doppler frames as well as camera frames are captured at the same sample rate, and the values given apply to both modalities.

Parameter	Value	Unit
Subjects	2	
Radar count	1	
Camera count	3	
Sample rate	25	FPS
Frames per clip	500	
Window size	192	frames
Window overlap	50	%
Total clips	178	
Total labeled windows	670	

**Table 3:** Class distribution of the soccer player action dataset used in this study. Given that the sensors are synchronized at the same sampling rate, these values correspond to both modalities.

Class	Segments	Frames	Windows
Walk	56	7720	116
Jog	78	6787	119
Dribble Walk	56	7863	118
Dribble Jog	70	7322	119
Jump	82	4220	56
Kick	48	2056	69
Crawl	30	5692	73
Total	420	41,660	670

**Table 4:** Radar feature groups extracted per frame.

Feature Group	Description
Global Doppler	Mean Doppler profile computed across all range bins.
Global Mel	Mel-filterbank energies [20].
Global MFCC	DCT of global mel energies [21].
Global Energy	Log-total mel energy representing motion intensity.
Block Doppler	Range-summed Doppler profile inside $B_{t}$.
Block Mel & MFCC	Mel and MFCC features computed on block Doppler.
Center Doppler	Doppler index of maximum energy inside the block.
Per-Range Mel	Mel energies computed independently for each block column (M×W matrix).

**Table 6:** Deep classifier architecture specifications. All models operate on sequences of shape (N,192,F), where *F* is the fused feature dimension.

Model	Type	Configuration	Notes
GRU	Recurrent	hidden = 128, layers = 2	Bidirectional; input LayerNorm; temporal mean pooling
LSTM	Recurrent	hidden = 128, layers = 2	Bidirectional; input LayerNorm; temporal mean pooling
CNN1D	Convolutional	128 → 128 → 256 ch	Kernel = 5; BatchNorm after each layer; AdaptiveAvgPool
TCN	Dilated conv.	(128, 128, 256, 256, 256) ch	Kernel = 5; dilation = $2^{i}$; causal; BatchNorm
CMT	Dual-stream	*d* = 256, heads = 8, layers = 2	Separate radar and camera encoders; bidirectional cross-attention

**Table 7:** Shared training hyperparameters for all deep classifiers.

Hyperparameter	Value
Optimizer	AdamW [36]
Learning rate	$5\times 10^{-4}$
Weight decay	$1\times 10^{-4}$
LR schedule	Cosine annealing [37], $T_{\max}=\max\left(10,\;\mathrm{epochs}\right)$
Batch size	16
Maximum epochs	60
Early stopping	Patience = 12 epochs (macro F1)
Loss function	Cross-entropy, inverse-frequency class weights
Gradient clipping	$\ell_{2}$ norm ≤1.0
Radar feature gate	Per-feature sigmoid, $\lambda_{L1}=1\times 10^{-3}$, init = 0.5
Mixed precision	FP16 autocast (GPU only)

**Table 8:** Single-feature results for classical and deep models using radar features. Values are mean ± std. Bold values indicate the best performance over all features and classifiers.

Feature	Classifier	acc	bAcc	F1_macro_
Block Center Doppler	LogReg	0.125 ± 0.033	0.152 ± 0.050	0.092 ± 0.032
	GRU	0.223 ± 0.036	0.253 ± 0.051	0.202 ± 0.028
	LSTM	0.221 ± 0.013	0.243 ± 0.017	0.200 ± 0.007
	1D-CNN	0.221 ± 0.037	0.232 ± 0.052	0.178 ± 0.043
	TCN	0.228 ± 0.025	0.252 ± 0.030	0.202 ± 0.024
Mel Spectrogram	LogReg	0.587 ± 0.049	0.631 ± 0.036	0.624 ± 0.039
	GRU	0.763 ± 0.033	0.786 ± 0.029	0.782 ± 0.036
	LSTM	0.731 ± 0.058	0.765 ± 0.047	0.756 ± 0.062
	1D-CNN	0.641 ± 0.055	0.673 ± 0.041	0.659 ± 0.056
	TCN	0.713 ± 0.040	0.744 ± 0.034	0.740 ± 0.045
MFCC	LogReg	0.594 ± 0.026	0.633 ± 0.018	0.626 ± 0.027
	GRU	0.788 ± 0.035	0.807 ± 0.031	0.804 ± 0.038
	LSTM	0.715 ± 0.043	0.744 ± 0.038	0.741 ± 0.046
	1D-CNN	0.713 ± 0.035	0.741 ± 0.040	0.735 ± 0.047
	TCN	0.756 ± 0.028	0.781 ± 0.013	0.777 ± 0.026
Block Spectrogram Per Range	LogReg	0.708 ± 0.053	0.706 ± 0.062	0.692 ± 0.062
	GRU	0.718 ± 0.066	0.719 ± 0.070	0.714 ± 0.074
	LSTM	0.753 ± 0.048	0.758 ± 0.060	0.750 ± 0.063
	1D-CNN	**0.783 ± 0.054**	0.785 ± 0.054	**0.785 ± 0.060**
	TCN	0.781 ± 0.058	**0.786 ± 0.060**	0.782 ± 0.076
Mel Energy	LogReg	0.167 ± 0.056	0.183 ± 0.059	0.148 ± 0.046
	GRU	0.347 ± 0.058	0.383 ± 0.050	0.364 ± 0.051
	LSTM	0.338 ± 0.068	0.383 ± 0.060	0.356 ± 0.056
	1D-CNN	0.286 ± 0.037	0.307 ± 0.055	0.272 ± 0.052
	TCN	0.361 ± 0.059	0.369 ± 0.057	0.359 ± 0.056

**Table 9:** Single-feature results for classical and deep models using camera features. Values are mean ± std. Bold values indicate the best performance over all features and classifiers.

Feature	Classifier	acc	bAcc	F1_macro_
HOG Full Frame	LogReg	0.977 ± 0.024	0.981 ± 0.020	0.981 ± 0.020
	GRU	0.983 ± 0.026	0.986 ± 0.021	0.985 ± 0.022
	LSTM	0.971 ± 0.020	0.976 ± 0.017	0.976 ± 0.017
	1D-CNN	**0.989 ± 0.016**	**0.990 ± 0.013**	**0.990 ± 0.013**
	TCN	**0.989 ± 0.016**	**0.990 ± 0.013**	**0.990 ± 0.013**
HOG ROI	LogReg	0.926 ± 0.056	0.939 ± 0.047	0.939 ± 0.044
	GRU	0.949 ± 0.055	0.952 ± 0.045	0.953 ± 0.045
	LSTM	0.954 ± 0.043	0.957 ± 0.035	0.957 ± 0.036
	1D-CNN	0.989 ± 0.026	0.990 ± 0.021	0.990 ± 0.022
	TCN	0.983 ± 0.016	0.986 ± 0.013	0.985 ± 0.014
Optical Flow	LogReg	0.714 ± 0.035	0.743 ± 0.043	0.733 ± 0.046
	GRU	0.891 ± 0.024	0.899 ± 0.028	0.892 ± 0.033
	LSTM	0.874 ± 0.059	0.887 ± 0.062	0.881 ± 0.069
	1D-CNN	0.880 ± 0.042	0.894 ± 0.041	0.886 ± 0.046
	TCN	0.920 ± 0.037	0.926 ± 0.034	0.925 ± 0.037
Bounding Box	LogReg	0.743 ± 0.083	0.782 ± 0.070	0.778 ± 0.071
	GRU	0.846 ± 0.043	0.865 ± 0.027	0.867 ± 0.028
	LSTM	0.829 ± 0.101	0.853 ± 0.077	0.847 ± 0.084
	1D-CNN	0.943 ± 0.020	0.953 ± 0.017	0.952 ± 0.017
	TCN	0.891 ± 0.055	0.903 ± 0.042	0.902 ± 0.046
Pose	LogReg	0.869 ± 0.059	0.868 ± 0.080	0.874 ± 0.072
	GRU	0.966 ± 0.024	0.966 ± 0.022	0.966 ± 0.022
	LSTM	0.971 ± 0.029	0.966 ± 0.033	0.968 ± 0.031
	1D-CNN	0.971 ± 0.035	0.971 ± 0.031	0.973 ± 0.031
	TCN	0.971 ± 0.029	0.970 ± 0.027	0.972 ± 0.026

**Table 10:** Feature omission (ablation) results for classical and deep models. Values are reported as mean ± standard deviation. The omitted feature whose removal causes the largest performance degradation is indicated in bold.

Omitted Feature	Classifier	acc	bAcc	F1_macro_
NONE	LogReg	0.982 ± 0.021	0.981 ± 0.027	0.981 ± 0.026
	GRU	0.990 ± 0.013	0.992 ± 0.010	0.991 ± 0.011
	LSTM	0.994 ± 0.008	0.995 ± 0.007	0.995 ± 0.007
	1D-CNN	0.996 ± 0.010	0.997 ± 0.007	0.996 ± 0.008
	TCN	0.996 ± 0.010	0.997 ± 0.007	0.996 ± 0.008
Radar Mel Spectrogram	LogReg	0.984 ± 0.022	0.982 ± 0.028	0.982 ± 0.027
	GRU	0.989 ± 0.014	0.991 ± 0.012	0.990 ± 0.013
	LSTM	0.994 ± 0.008	0.995 ± 0.007	0.995 ± 0.007
	1D-CNN	0.994 ± 0.009	0.996 ± 0.007	0.995 ± 0.008
	TCN	0.996 ± 0.010	0.997 ± 0.007	0.996 ± 0.008
Radar MFCC	LogReg	0.979 ± 0.024	0.979 ± 0.029	0.979 ± 0.028
	GRU	0.991 ± 0.012	0.992 ± 0.010	0.992 ± 0.011
	LSTM	0.995 ± 0.007	0.995 ± 0.006	0.996 ± 0.006
	1D-CNN	0.996 ± 0.010	0.997 ± 0.007	0.996 ± 0.008
	TCN	0.996 ± 0.010	0.997 ± 0.007	0.996 ± 0.008
Radar Block Spectrogram Per Range	LogReg	0.993 ± 0.010	0.994 ± 0.008	0.994 ± 0.009
	GRU	0.988 ± 0.016	0.991 ± 0.012	0.990 ± 0.013
	LSTM	0.992 ± 0.010	0.993 ± 0.008	0.993 ± 0.009
	1D-CNN	0.996 ± 0.010	0.997 ± 0.007	0.997 ± 0.008
	TCN	0.996 ± 0.010	0.997 ± 0.007	0.996 ± 0.008
Camera HOG Full Frame	LogReg	**0.912 ± 0.038**	**0.920 ± 0.042**	**0.918 ± 0.043**
	GRU	0.983 ± 0.026	0.986 ± 0.021	0.985 ± 0.022
	LSTM	0.971 ± 0.020	0.976 ± 0.017	0.976 ± 0.017
	1D-CNN	0.988 ± 0.011	0.987 ± 0.015	0.988 ± 0.013
	TCN	0.989 ± 0.016	0.990 ± 0.013	0.990 ± 0.013
Camera HOG ROI	LogReg	0.959 ± 0.033	0.966 ± 0.027	0.965 ± 0.028
	GRU	0.983 ± 0.026	0.986 ± 0.021	0.985 ± 0.022
	LSTM	0.971 ± 0.020	0.976 ± 0.017	0.976 ± 0.017
	1D-CNN	0.991 ± 0.010	0.992 ± 0.008	0.992 ± 0.008
	TCN	0.983 ± 0.016	0.986 ± 0.013	0.985 ± 0.014
Camera Optical Flow	LogReg	0.982 ± 0.021	0.981 ± 0.027	0.981 ± 0.026
	GRU	0.983 ± 0.026	0.986 ± 0.021	0.985 ± 0.022
	LSTM	0.971 ± 0.020	0.976 ± 0.017	0.976 ± 0.017
	1D-CNN	0.994 ± 0.009	0.996 ± 0.007	0.995 ± 0.008
	TCN	0.996 ± 0.010	0.997 ± 0.007	0.997 ± 0.008
Camera Bounding Box	LogReg	0.981 ± 0.021	0.980 ± 0.026	0.980 ± 0.026
	GRU	0.983 ± 0.026	0.986 ± 0.021	0.985 ± 0.022
	LSTM	0.971 ± 0.020	0.976 ± 0.017	0.976 ± 0.017
	1D-CNN	0.996 ± 0.010	0.997 ± 0.007	0.996 ± 0.008
	TCN	0.996 ± 0.010	0.997 ± 0.007	0.996 ± 0.008
Camera Pose	LogReg	0.982 ± 0.021	0.981 ± 0.027	0.981 ± 0.026
	GRU	0.983 ± 0.026	0.986 ± 0.021	0.985 ± 0.022
	LSTM	0.971 ± 0.020	0.976 ± 0.017	0.976 ± 0.017
	1D-CNN	0.996 ± 0.010	0.997 ± 0.007	0.997 ± 0.008
	TCN	0.996 ± 0.010	0.997 ± 0.007	0.996 ± 0.008

**Table 11:** Radar only results for jog, walk, dribble jog, dribble walk, and crawl for the different movement directions using log regression, GRU, LSTM, 1D-CNN, and TCN.

Direction	Classifier	acc	bAcc	F1_macro_	Precision	Recall
Tangential	LogReg	0.614 ± 0.245	0.624 ± 0.218	0.563 ± 0.270	0.560 ± 0.294	0.624 ± 0.218
	GRU	0.609 ± 0.298	0.613 ± 0.266	0.555 ± 0.318	0.563 ± 0.321	0.613 ± 0.266
	LSTM	0.704 ± 0.248	0.704 ± 0.226	0.646 ± 0.278	0.653 ± 0.298	0.704 ± 0.226
	1D-CNN	0.785 ± 0.147	0.772 ± 0.135	0.761 ± 0.141	0.812 ± 0.103	0.772 ± 0.135
	TCN	0.736 ± 0.136	0.708 ± 0.097	0.666 ± 0.107	0.712 ± 0.122	0.708 ± 0.097
Radial	LogReg	0.787 ± 0.177	0.790 ± 0.168	0.763 ± 0.199	0.756 ± 0.206	0.790 ± 0.168
	GRU	0.860 ± 0.107	0.855 ± 0.133	0.827 ± 0.165	0.856 ± 0.145	0.855 ± 0.133
	LSTM	0.802 ± 0.139	0.805 ± 0.144	0.791 ± 0.146	0.814 ± 0.114	0.805 ± 0.144
	1D-CNN	0.925 ± 0.090	0.930 ± 0.076	0.925 ± 0.085	0.940 ± 0.068	0.930 ± 0.076
	TCN	0.882 ± 0.073	0.885 ± 0.072	0.879 ± 0.073	0.889 ± 0.071	0.885 ± 0.072
Diagonal	LogReg	0.678 ± 0.143	0.700 ± 0.134	0.678 ± 0.139	0.699 ± 0.161	0.700 ± 0.134
	GRU	0.688 ± 0.097	0.716 ± 0.084	0.694 ± 0.088	0.709 ± 0.097	0.716 ± 0.084
	LSTM	0.686 ± 0.131	0.710 ± 0.128	0.691 ± 0.122	0.732 ± 0.137	0.710 ± 0.128
	1D-CNN	0.858 ± 0.057	0.871 ± 0.047	0.862 ± 0.050	0.873 ± 0.053	0.871 ± 0.047
	TCN	0.822 ± 0.054	0.838 ± 0.059	0.822 ± 0.060	0.842 ± 0.055	0.838 ± 0.059
Horizontal	LogReg	0.477 ± 0.178	0.477 ± 0.159	0.433 ± 0.166	0.472 ± 0.205	0.477 ± 0.159
	GRU	0.496 ± 0.090	0.524 ± 0.109	0.495 ± 0.100	0.525 ± 0.062	0.524 ± 0.109
	LSTM	0.642 ± 0.115	0.661 ± 0.107	0.614 ± 0.124	0.674 ± 0.141	0.661 ± 0.107
	1D-CNN	0.856 ± 0.078	0.851 ± 0.095	0.849 ± 0.082	0.887 ± 0.057	0.851 ± 0.095
	TCN	0.821 ± 0.112	0.814 ± 0.136	0.803 ± 0.150	0.812 ± 0.157	0.814 ± 0.136
Combined	LogReg	0.780 ± 0.083	0.784 ± 0.081	0.775 ± 0.085	0.786 ± 0.086	0.784 ± 0.081
	GRU	0.717 ± 0.059	0.726 ± 0.045	0.712 ± 0.060	0.729 ± 0.072	0.726 ± 0.045
	LSTM	0.723 ± 0.079	0.726 ± 0.082	0.726 ± 0.076	0.740 ± 0.072	0.726 ± 0.082
	1D-CNN	0.819 ± 0.107	0.825 ± 0.109	0.825 ± 0.105	0.843 ± 0.095	0.825 ± 0.109
	TCN	0.859 ± 0.029	0.862 ± 0.034	0.860 ± 0.034	0.876 ± 0.030	0.862 ± 0.034

**Table 12:** Classification results achieved for different combinations of a single modality for all classes and directions using log regression, GRU, LSTM, 1D-CNN, and TCN. Bold values indicate the best performance per modality.

Sensor	Classifier	acc	bAcc	F1_macro_	Precision	Recall
Radar	LogReg	0.742 ± 0.028	0.744 ± 0.031	0.736 ± 0.025	0.754 ± 0.031	0.744 ± 0.031
	GRU	0.721 ± 0.061	0.737 ± 0.056	0.730 ± 0.060	0.742 ± 0.062	0.737 ± 0.056
	LSTM	0.701 ± 0.035	0.706 ± 0.043	0.702 ± 0.042	0.731 ± 0.031	0.706 ± 0.043
	1D-CNN	0.865 ± 0.055	0.876 ± 0.054	0.873 ± 0.048	0.878 ± 0.043	0.876 ± 0.054
	TCN	**0.886 ± 0.034**	**0.901 ± 0.028**	**0.897 ± 0.033**	**0.903 ± 0.035**	**0.901 ± 0.028**
Center Cam	LogReg	0.956 ± 0.035	0.966 ± 0.027	0.964 ± 0.028	0.965 ± 0.026	0.966 ± 0.027
	GRU	0.985 ± 0.026	0.989 ± 0.019	0.988 ± 0.020	0.990 ± 0.015	0.989 ± 0.019
	LSTM	0.979 ± 0.035	0.984 ± 0.026	0.983 ± 0.028	0.985 ± 0.024	0.984 ± 0.026
	1D-CNN	0.988 ± 0.023	0.991 ± 0.018	0.990 ± 0.018	0.991 ± 0.017	0.991 ± 0.018
	TCN	**0.992 ± 0.017**	**0.994 ± 0.013**	**0.993 ± 0.015**	**0.993 ± 0.015**	**0.994 ± 0.013**
Left Cam	LogReg	0.978 ± 0.019	0.980 ± 0.014	0.978 ± 0.015	0.977 ± 0.014	0.980 ± 0.014
	GRU	0.985 ± 0.016	0.987 ± 0.013	0.984 ± 0.016	0.984 ± 0.017	0.987 ± 0.013
	LSTM	0.988 ± 0.014	0.990 ± 0.011	0.988 ± 0.014	0.988 ± 0.016	0.990 ± 0.011
	1D-CNN	**0.994 ± 0.010**	**0.994 ± 0.008**	0.993 ± 0.012	0.993 ± 0.014	**0.994 ± 0.008**
	TCN	0.993 ± 0.009	0.993 ± 0.008	**0.993 ± 0.008**	**0.994 ± 0.008**	0.993 ± 0.008
Right Cam	LogReg	0.972 ± 0.019	0.975 ± 0.016	0.975 ± 0.016	0.977 ± 0.014	0.975 ± 0.016
	GRU	0.994 ± 0.010	0.994 ± 0.008	0.994 ± 0.008	0.995 ± 0.008	0.994 ± 0.008
	LSTM	0.993 ± 0.013	0.994 ± 0.010	0.994 ± 0.010	0.995 ± 0.009	0.994 ± 0.010
	1D-CNN	**0.997 ± 0.007**	**0.998 ± 0.006**	**0.997 ± 0.007**	**0.997 ± 0.007**	**0.998 ± 0.006**
	TCN	0.994 ± 0.010	0.994 ± 0.008	0.995 ± 0.008	0.996 ± 0.007	0.994 ± 0.008
Right + Center Cam	LogReg	0.976 ± 0.013	0.980 ± 0.010	0.980 ± 0.010	0.981 ± 0.010	0.980 ± 0.010
	GRU	0.994 ± 0.010	0.995 ± 0.008	0.995 ± 0.009	0.995 ± 0.009	0.995 ± 0.008
	LSTM	0.989 ± 0.011	0.991 ± 0.009	0.991 ± 0.010	0.991 ± 0.010	0.991 ± 0.009
	1D-CNN	**0.995 ± 0.010**	**0.996 ± 0.008**	**0.996 ± 0.008**	**0.997 ± 0.007**	**0.996 ± 0.008**
	TCN	**0.995 ± 0.010**	**0.996 ± 0.008**	**0.996 ± 0.008**	**0.997 ± 0.007**	**0.996 ± 0.008**
Left + Right + Center Cam	LogReg	0.993 ± 0.011	0.994 ± 0.009	0.994 ± 0.009	0.994 ± 0.008	0.994 ± 0.009
	GRU	**0.997 ± 0.004**	**0.998 ± 0.003**	**0.997 ± 0.004**	**0.997 ± 0.005**	**0.998 ± 0.003**
	LSTM	0.996 ± 0.007	0.996 ± 0.005	0.996 ± 0.005	**0.997 ± 0.005**	0.996 ± 0.005
	1D-CNN	0.995 ± 0.010	0.996 ± 0.008	0.996 ± 0.008	0.997 ± 0.007	0.996 ± 0.008
	TCN	0.994 ± 0.010	0.995 ± 0.008	0.995 ± 0.009	0.995 ± 0.009	0.995 ± 0.008

**Table 13:** Classification results obtained for the fusion of different modalities for all classes and directions using log regression, GRU, LSTM, 1D-CNN, TCN, and CMT. Bold values indicate the best performance per modality.

Sensor	Classifier	acc	bAcc	F1_macro_	Precision	Recall
Radar + Center Cam	LogReg Early	0.943 ± 0.038	0.954 ± 0.029	0.952 ± 0.032	0.954 ± 0.031	0.954 ± 0.029
	LogReg Late	0.959 ± 0.031	0.968 ± 0.024	0.966 ± 0.025	0.968 ± 0.023	0.968 ± 0.024
	GRU	0.984 ± 0.029	0.987 ± 0.023	0.987 ± 0.023	0.988 ± 0.022	0.987 ± 0.023
	LSTM	0.985 ± 0.030	0.988 ± 0.023	0.988 ± 0.023	0.988 ± 0.023	0.988 ± 0.023
	1D-CNN	**0.994 ± 0.013**	**0.995 ± 0.011**	**0.995 ± 0.011**	**0.996 ± 0.009**	**0.995 ± 0.011**
	TCN	0.991 ± 0.016	0.993 ± 0.012	0.992 ± 0.014	0.992 ± 0.014	0.993 ± 0.012
	CMT	0.988 ± 0.014	0.990 ± 0.012	0.990 ± 0.012	0.991 ± 0.011	0.990 ± 0.012
Radar + Right + Center Cam	LogReg Early	0.973 ± 0.025	0.979 ± 0.020	0.978 ± 0.021	0.979 ± 0.020	0.979 ± 0.020
	LogReg Late	0.972 ± 0.011	0.976 ± 0.008	0.976 ± 0.009	0.978 ± 0.009	0.976 ± 0.008
	GRU	0.990 ± 0.013	0.991 ± 0.010	0.992 ± 0.010	0.993 ± 0.009	0.991 ± 0.010
	LSTM	0.989 ± 0.020	0.992 ± 0.015	0.991 ± 0.016	0.991 ± 0.016	0.992 ± 0.015
	1D-CNN	0.995 ± 0.010	0.996 ± 0.008	0.996 ± 0.008	0.997 ± 0.007	0.996 ± 0.008
	TCN	0.995 ± 0.010	0.996 ± 0.008	0.996 ± 0.008	0.997 ± 0.007	0.996 ± 0.008
	CMT	**0.997 ± 0.007**	**0.998 ± 0.005**	**0.998 ± 0.005**	**0.998 ± 0.005**	**0.998 ± 0.005**
Radar + Left + Right + Center Cam	LogReg Early	0.987 ± 0.016	0.989 ± 0.013	0.989 ± 0.013	0.990 ± 0.012	0.989 ± 0.013
	LogReg Late	0.993 ± 0.011	0.994 ± 0.009	0.994 ± 0.009	0.994 ± 0.008	0.994 ± 0.009
	GRU	0.994 ± 0.010	0.995 ± 0.008	0.995 ± 0.009	0.995 ± 0.009	0.995 ± 0.008
	LSTM	0.992 ± 0.009	0.994 ± 0.008	0.993 ± 0.009	0.993 ± 0.009	0.994 ± 0.008
	1D-CNN	0.997 ± 0.007	0.998 ± 0.006	0.997 ± 0.007	0.997 ± 0.007	0.998 ± 0.006
	TCN	0.995 ± 0.010	0.996 ± 0.008	0.996 ± 0.009	0.996 ± 0.009	0.996 ± 0.008
	CMT	**0.997 ± 0.007**	**0.998 ± 0.005**	**0.998 ± 0.005**	**0.998 ± 0.005**	**0.998 ± 0.005**

**Table 14:** Duration and frame rate of radar and camera data retrieval and pre-processing.

Stage	Modality	Frame Proc. Time (ms)	FPS
Load ADC data (CPU)	Radar	13.021	76.799
ADC → Range–Doppler (GPU)	Radar	3.010	332.226
Video decoding (CPU)	Camera	1.891	528.821

**Table 15:** Computational cost of radar and camera feature extraction stages. The normalized processing time is given as a percentage of the longest processing time. The radar processing was primarily run on an Nvidia RTX3060 GPU, whereas the camera processing was primarily performed on an Intel Core i5-8500 CPU. For the camera processing, only YOLO detections were performed using a GPU, which negatively impacts the processing times.

Stage	Modality	Time per Frame (ms)	FPS	Normalized Proc. Time (%)
CFAR mask (detection)	Radar	1.684	593.824	1.79
Mel filterbank	Radar	3.356	297.974	3.56
Block localization	Radar	0.633	1579.779	0.67
Global spectrogram mean	Radar	0.274	3649.635	0.29
Block spectrogram	Radar	0.270	3703.704	0.29
Mel spectrogram (global + block)	Radar	1.133	882.613	1.20
MFCC extraction	Radar	2.134	468.604	2.27
Object detection and bounding box	Camera	94.189	10.617	100
HOG features (full frame)	Camera	0.618	1618.123	0.66
HOG features (ROI)	Camera	0.543	1841.621	0.58
Optical flow	Camera	4.017	248.942	4.26
Pose estimation	Camera	29.188	34.261	30.99

**Table 16:** Training and classification time across modalities for the full training and test set. All values are in seconds.

Mode	PCA	LogReg	GRU	LSTM	1D-CNN	TCN	CMT
Radar only	7.664	80.56	276.03	168.37	242.35	265.28	-
Single camera only	2.810	49.97	43.35	40.99	35.24	67.24	-
All cameras only	5.645	57.20	74.33	81.42	76.34	93.66	-
Radar + single camera	4.053	82.5	71.25	71.62	72.51	88.87	96.98
Radar + all cameras	28.030	97.32	155.94	151.52	142.01	184.41	194.82

## Data Availability

The dataset will be made publicly available within 3 months of publication through the University of Pretoria research data repository, in accordance with ethical approval conditions. The dataset will include synchronized radar range–Doppler cubes and multiview camera recordings, along with the corresponding annotations and documentation. To support reproducibility, the core implementation of the proposed framework will be made publicly available via a GitHub repository within the same timeframe. The repository will include the radar processing pipeline, feature extraction methods, and classification framework required to reproduce the reported results.

## Abbreviations

The following abbreviations are used in this manuscript:

CFAR	constant false alarm rate
CMT	cross modal transformer
CNN	convolutional neural network
FPS	frames per second
GMM-HMM	Gaussian mixture Hidden Markov model
GRU	gated recurrent network
HAR	human action recognition
HOG	histogram of oriented gradients
IMU	inertial measurement unit
LSTM	long short-term memory
MFCC	mel-frequency cepstral coefficients
PCA	principal component analysis
ROI	region of interest
t-SNE	t-distributed stochastic neighbor embedding
TCN	temporal convolutional network
